# Astrocytic BDNF and TrkB regulate severity and neuronal activity in mouse models of temporal lobe epilepsy

**DOI:** 10.1038/s41419-020-2615-9

**Published:** 2020-06-01

**Authors:** Sara Fernández-García, Anna Sancho-Balsells, Sophie Longueville, Denis Hervé, Agnès Gruart, José María Delgado-García, Jordi Alberch, Albert Giralt

**Affiliations:** 1 Departament de Biomedicina, Facultat de Medicina, Institut de Neurociències, Universitat de Barcelona, 08036 Barcelona, Spain; 2 Institut d’Investigacions Biomèdiques August Pi i Sunyer (IDIBAPS), 08036 Barcelona, Spain; 3 Centro de Investigación Biomédica en Red sobre Enfermedades Neurodegenerativas (CIBERNED), 28031 Madrid, Spain; 4 Inserm UMR-S 1270, 75005 Paris, France; 5 Sorbonne Université, Science and Engineering Faculty, 75005 Paris, France; 6 Institut du Fer a Moulin, 75005 Paris, France; 7 Division of Neurosciences, Pablo de Olavide University, 41013 Seville, Spain; 8 Production and Validation Center of Advanced Therapies (Creatio), Faculty of Medicine and Health Science, University of Barcelona, 08036 Barcelona, Spain

**Keywords:** Neuroscience, Epilepsy

## Abstract

Astrocytes have emerged as crucial regulators of neuronal network activity, synapse formation, and underlying behavioral and cognitive processes. Despite some pathways have been identified, the communication between astrocytes and neurons remains to be completely elucidated. Unraveling this communication is crucial to design potential treatments for neurological disorders like temporal lobe epilepsy (TLE). The BDNF and TrkB molecules have emerged as very promising therapeutic targets. However, their modulation can be accompanied by several off-target effects such as excitotoxicity in case of uncontrolled upregulation or dementia, amnesia, and other memory disorders in case of downregulation. Here, we show that BDNF and TrkB from astrocytes modulate neuronal dysfunction in TLE models. First, conditional overexpression of BDNF from astrocytes worsened the phenotype in the lithium-pilocarpine mouse model. Our evidences pointed out to the astrocytic pro-BDNF isoform as a major player of this altered phenotype. Conversely, specific genetic deletion of BDNF in astrocytes prevented the increase in the number of firing neurons and the global firing rate in an in vitro model of TLE. Regarding to the TrkB, we generated mice with a genetic deletion of TrkB specifically in hippocampal neurons or astrocytes. Interestingly, both lines displayed neuroprotection in the lithium-pilocarpine model but only the mice with genetic deletion of TrkB in astrocytes showed significantly preserved spatial learning skills. These data identify the astrocytic BDNF and TrkB molecules as promising therapeutic targets for the treatment of TLE.

## Introduction

Correct function of neuronal networks depends on an appropriate modulation by astrocytes^1^. Activated astrocytes provide both excitatory and inhibitory effects on neighboring neurons^2^. Thus, astrocytes can modulate neuronal activity and, in turn, neurons exert functional changes in astrocytes upon neural activity such as modifications in gene expression, gliotransmitter release and intracellular calcium signals^3–6^. However, mechanisms underlying astrocyte–neuron reciprocal communication remain poorly understood.

Recent literature has shown that BDNF and TrkB are expressed in both neurons and astrocytes^7^. This expression pattern suggests that the BDNF–TrkB pathway could be a core system modulating astrocyte–neuron reciprocal communication. The neurotrophin BDNF is a key player in learning and memory processes, synaptic plasticity phenomena, and neuronal survival^8,9^ as well as TrkB, its main receptor^10,11^. However, BDNF and TrkB molecules have often opposed biological effects explained by the presence of the different TrkB isoforms and the antagonistic biological activity of mature (mBDNF) and precursor (pro-BDNF) forms of BDNF^12^. As an example, pro-BDNF can activate p75^NTR^ to induce neurotoxicity whereas mBDNF can activate TrkB to induce neuroplasticity^12^. On the other hand, the truncated isoform of TrkB receptor (TrkB.T1) disrupts/counteracts the full-length (TrkB.FL) active receptor function^13,14^ by promoting the formation of non-functional heterodimers^15^. In contrast to the TrkB.FL-dependent signaling, TrkB.T1-mediated signaling has been suggested to mediate neurodegenerative processes in excitotoxic contexts^16^. Thus, excess of (pro)BDNF or TrkB.T1 protein levels as well as aberrant interaction between TrkB and p75^NTR^ receptors may lead to neuronal dysfunction or death via induction of excitotoxicity-related molecular pathways^12,17^.

A detrimental role of TrkB isoforms has been observed in temporal lobe epilepsy (TLE) models^18^. In particular, the increase in TrkB.T1 protein levels could be one of the underlying molecular mechanisms of neuronal death in TLE models^19^. However, whether astrocytes are regulating synaptic excitability or activation of neuronal populations in TLE-associated neurodegeneration via the BDNF and TrkB molecules remains elusive.

In the present work we show that BDNF overexpression in astrocytes leads to enhanced excitability and neuronal cell death. Furthermore, we show that BDNF from astrocytes is necessary to increase the number of firing neurons in an in vitro model of TLE. In a second subset of results exploring the role of the astrocytic TrkB receptor, we show that a knockdown of total TrkB in astrocytes is beneficial in TLE. Concretely, we show that TrkB deletion in hippocampal astrocytes is neuroprotective in TLE models and preserves with higher rates hippocampal-related cognitive functions when compared to the genetic deletion of TrkB in neurons.

## Results

### pGFAP-BDNF mice display worse phenotype and increased neuronal death in the lithium-pilocarpine model of TLE

**Fig. 1:**
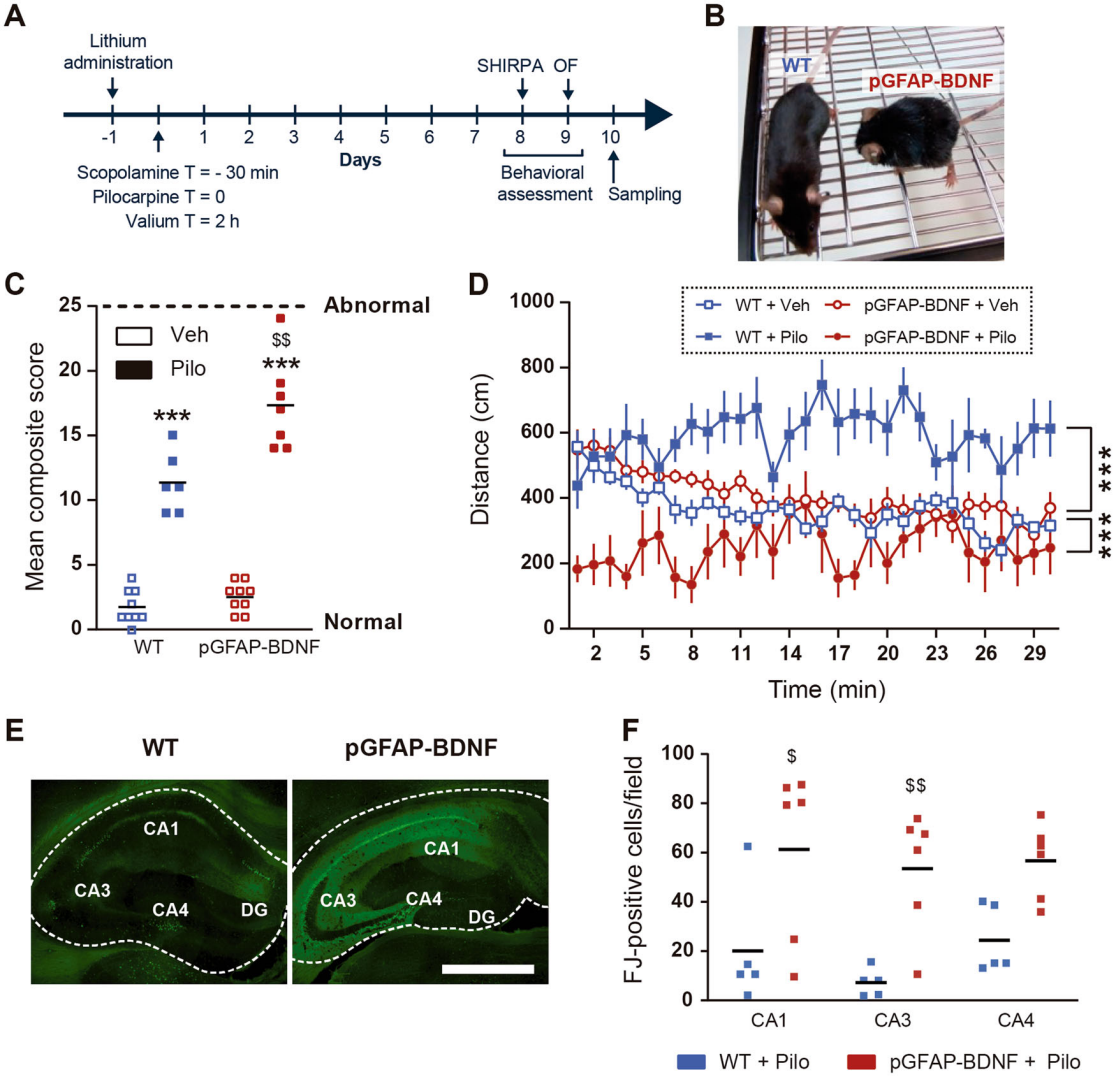
Effects of lithium pilocarpine in the pGFAP-BDNF mice. **a** Schematic representation of the experimental design. **b** Representative picture showing WT (left) and pGFAP-BDNF (right) mice 10 days after pilocarpine (Pilo) treatment. **c** All the items of the SHIRPA battery test are pulled as a mean composite score. Statistical analysis indicate that pilocarpine induces a significant increase in abnormal scores (two-way ANOVA, variable treatment: *F*_(3,29)_ = 79.71; *p* < 0.001) and that pGFAP-BDNF mice exhibit the worse score (post hoc test, *p* < 0.001). **d** Pathlength in a 30-min open-field session is depicted. Results indicate strong significant differences between groups (two-way ANOVA, variable treatment: *F*_(3,27)_ = 19.81, *p* < 0.001). WT + Pilo mice displayed a significant increase on locomotor activity compared to WT + Veh (post hoc test: *p* < 0.001) whereas pGFAP-BDNF + Pilo mice displayed a significant decrease on locomotor activity compared to pGFAP-BDNF + Veh mice (post hoc test: *p* < 0.001). **e** Fluoro-Jade staining was performed 10 days after pilocarpine treatment. Photomicrographs show the hippocampal areas positive for Fluoro-Jade in WT (left image) and pGFAP-BDNF (right image) mice treated with pilocarpine. Scale bar, 500 μm. **f** Graph showing the quantification of the Fluoro-Jade-positive cells per field in CA1, CA3, and CA4. Statistical analysis indicated that pGFAP-BDNF mice showed significant higher rates of neuronal cell death than WT mice when treated with pilocarpine (two-way ANOVA, variable genotype: *F*_(1,27)_ = 26.42, *p* < 0.001). Bars represent mean ± SEM. Data were analyzed by two-way analysis of variance (ANOVA) with Bonferroni’s test as a post hoc. *p < 0.05 and ***p < 0.001 when compared with vehicle controls; ^$^*p* < 0.05 and ^$$^*p* < 0.01 when compared with WT + Pilo. For behavioral experiments: WT + Veh (*n* = 9), WT + Pilo (*n* = 6), pGFAP-BDNF + Veh (*n* = 9), and pGFAP-BDNF + Pilo (*n* = 7). For biochemical experiments: WT + Veh (*n* = 5), WT + Pilo (*n* = 7), pGFAP-BDNF + Veh (*n* = 6), and pGFAP-BDNF + Pilo (*n* = 8). For the Fluoro-Jade experiment: WT + Pilo (*n* = 5) and pGFAP-BDNF + Pilo (*n* = 6). All mice were used at 10 weeks of age and were males.

First, since it has been widely demonstrated that BDNF is expressed and released by astrocytes^20–23^, we aimed to study the role of the neurotrophin produced by astrocytes in a TLE model by using wild-type (WT) mice and the pGFAP-BDNF transgenic mice as a gain-of-BDNF-function approach. In these mice, BDNF is overexpressed only under pathological conditions^20,21^. To do so, first we subjected pGFAP-BDNF mice and their WT controls to the lithium-pilocarpine model (Fig. 1a). Next, mice were subjected to the SHIRPA and open-field paradigms 8–9 days later (Fig. 1a). At day 8 after the pilocarpine injection, pGFAP-BDNF mice displayed a general visually worse state than WT mice with more curved position and groomed fur (Fig. 1b). The SHIRPA protocol, which is a rapid, comprehensive screening method for qualitatively assess abnormal phenotypes of the mouse neurological state and health^24^ was performed in these mice. Thus, the SHIRPA general index (Fig. 1c) demonstrated that pGFAP-BDNF mice exhibited a significantly worse neurological state than WT mice when treated with pilocarpine. We next observed in the open-field (OF) paradigm that WT mice treated with pilocarpine displayed a significant increase in their pathlength compared to WT and pGFAP-BDNF mice treated with vehicle (Fig. 1d). Conversely, pGFAP-BDNF mice treated with pilocarpine showed a significant reduction on their pathlength compared with WT and pGFAP-BDNF mice treated with vehicle according to their worse neurological state. Then, we evaluated hippocampal neuronal death by performing a Fluoro-Jade staining in the same mice after 10 days of pilocarpine treatment. We found that neuronal death was significantly more prominent in pGFAP-BDNF mice than in WT mice specifically in the CA1 and CA3 but not in the CA4 (Fig. 1e, f).

**Fig. 2:**
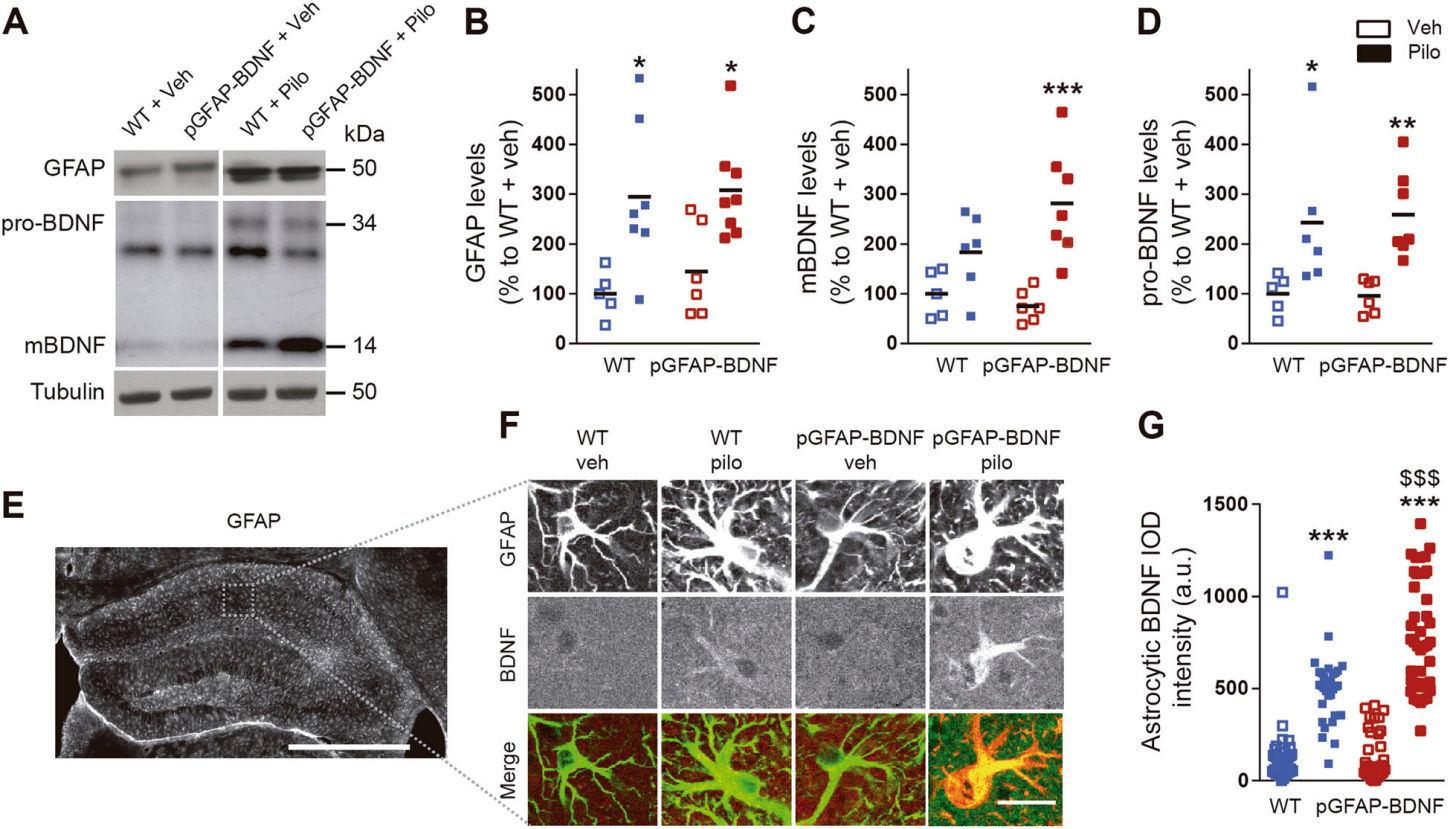
Astrocytic BDNF levels in the hippocampus of the pilocarpine-treated WT and pGFAP-BDNF mice. **a** Immunoblotting analysis of GFAP, BDNF, and tubulin as a loading control in WT and pGFAP-BDNF mice treated with vehicle or pilocarpine. **b** Densitometry quantification of GFAP results as in **a**. Statistical analysis revealed a significant increase on GFAP protein levels in groups of mice treated with pilocarpine (two-way ANOVA, variable treatment: *F*_(1,22)_ = 17.44, *p* < 0.001). **c** Densitometry quantification of the mature form of BDNF (mBDNF) results as in **a**. Statistical analysis revealed a significant increase on mBDNF protein levels (two-way ANOVA variable treatment: *F*_(1,26)_ = 16.89, *p* < 0.001) specifically in pGFAP-BDNF mice treated with pilocarpine (post hoc test: *p* < 0.001). **d** Densitometry quantification of pro-BDNF results as in **a**. Statistical analysis revealed a significant increase on pro-BDNF protein levels (two-way ANOVA variable treatment: *F*_(1,20)_ = 17.50, *p* < 0.001) in both WT and pGFAP-BDNF mice treated with pilocarpine. **e** Representative image showing GFAP immunofluorescence in the hippocampus. Scale bar, 500 μm. **f** Representative immunofluorescence images showing GFAP and BDNF immunoreactivity in the hippocampus of WT and pGFAP-BDNF mice treated with vehicle or pilocarpine. Scale bar, 20 µm. **g** Quantification of astrocytic BDNF immunoreactivity. Statistical analysis revealed a significant increase in the astrocytic BNDF (two-way ANOVA interaction: *F*_(1,149)_ = 16.37, *p* < 0.0001; variable treatment: *F*_(1,149)_ = 235.8, *p* < 0.0001; variable genotype: *F*_(1,149)_ = 20.55, *p* < 0.0001). Data were analyzed by two-way ANOVA followed by Bonferroni’s test. *p < 0.05, **p < 0.01, ***p < 0.001 compared to Veh. ^$$$^*p* < 0.001 compared to WT-Pilo. All mice were used at 10 weeks of age and were males.

To validate the model, we confirmed that both, WT and pGFAP-BDNF, mice showed significant increased levels of GFAP after pilocarpine treatment (Fig. 2a, b). These results were accompanied by a significant increase of pro-BDNF levels in both groups and with a specific increase of mature BDNF only in pGFAP-BDNF mice but not in WT mice (Fig. 2a, c, d). This result does not rule out the possibility that BDNF levels were also increased in WT mice. To tackle this question, we performed an immunofluorescence against BDNF in fixed slices from the same mice. We observed a specific increase in the BDNF immunoreactivity in hippocampal WT and pGFAP-BDNF astrocytes localized in the *stratum radiatum* of the CA1 (Fig. 2e–g). Furthermore, this pilocarpine-dependent increase of BDNF immunoreactivity was significantly higher in pGFAP-BDNF astrocytes than in WT astrocytes (Fig. 2f, g). Taken together, these results indicated that, in physiological conditions, the pro-BDNF isoform was aberrantly increased in a sustained manner in astrocytes 10 days after the pilocarpine treatment and that the worse phenotype observed in the pGFAP-BDNF mice correlated with a specific increase of the mature form of BDNF.

### Hippocampal CA3–CA1 excitability is increased in pGFAP-BDNF mice

**Fig. 3:**
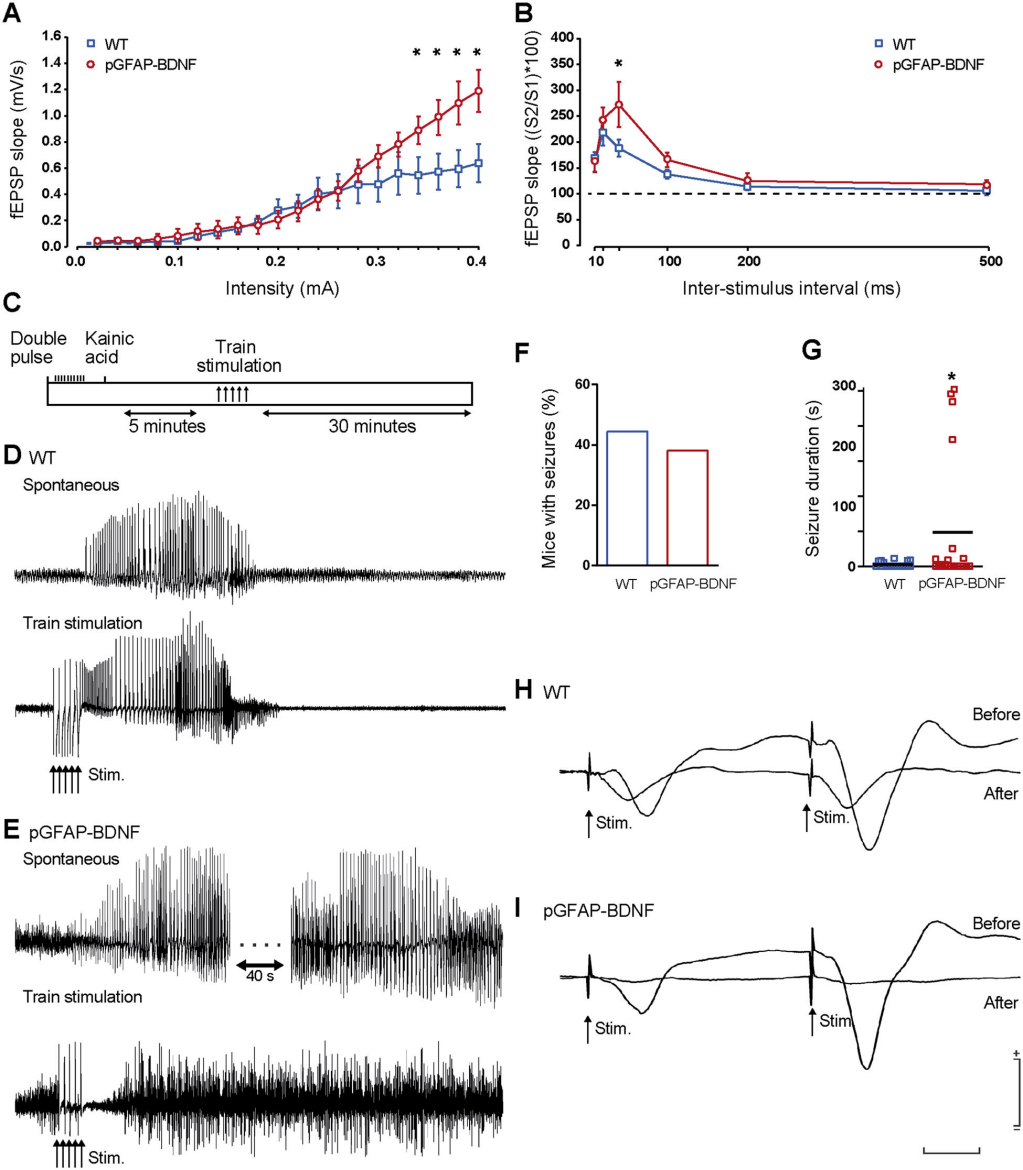
Basic functional properties of CA3–CA1 synapses and effects of kainic acid administration in WT and pGFAP-BDNF mice. **a** Input/output curves evoked in a new cohort of WT and pGFAP-BDNF mice that did not received the lithium-pilocarpine treatment. Note that the pGFAP-BDNF group reached significantly larger fEPSP slopes for high (<0.3 mA) stimulus intensities. *p < 0.05. **b** fEPSP facilitation evoked at the hippocampal CA1 area by paired-pulse stimulation of ipsilateral Schaffer collaterals at increasing interstimulus intervals. Note the significant (*p = 0.003) increase in paired-pulse facilitation at 40 ms of interstimulus interval in pGFAP-BDNF mice. **c** A diagram illustrating the experimental design for the kainic acid test. **d**, **e** Representative examples of spontaneous and train-evoked seizures collected from WT (**d**) and pGFAP-BDNF (**e**) mice. Note the different duration of tonic seizures presented by the two groups of animals. **f** Percentage of animals presenting seizures following kainate injection. **g** Mean duration of tonic seizures recorded in WT and pGFAP-BDNF mice. *p = 0.003. **h**, **i** Representative examples of fEPSPs evoked at hippocampal CA3–CA1 synapses and collected from WT (**h**) and pGFAP-BDNF (**i**) mice before and after kainic acid injection. Note that the presence of tonic seizures decreased in the amplitude of the evoked fEPSP in the pGFAP-BDNF mouse.

In a new cohort of WT and pGFAP-BDNF mice (without any previous treatment with pilocarpine), we recorded input/output curves and paired-pulse facilitation at the CA3–CA1 synapse in the two groups of mice in vivo (Fig. 3a, b). The two groups of mice (*n* = 10/group) increased significantly in field excitatory postsynaptic potentials (fEPSP) slopes with the increase in stimulus intensities (*F*_(19,399)_ = 40.916; *p* < 0.001). Slopes of fEPSPs evoked in pGFAP-BDNF mice were significantly larger than those presented by WT animals for higher stimulus intensities (≥0.34 mA; *F*_(19,399)_ = 4.023; *p* ≤ 0.05) (Fig. 3a). Altogether suggests a higher excitability of hippocampal CA3–CA1 synapses in pGFAP-BDNF mice.

Changes in synaptic strength evoked by the presentation of a pair of pulses are a form of pre-synaptic short-term plasticity^25^. The two groups of mice presented a paired-pulse facilitation at short (20 and 40 ms) inter-pulse intervals (*F*_(5,119)_ = 19.025; *p* < 0.001). Nevertheless, the pGFAP-BDNF group presented a significantly larger paired-pulse facilitation at the interval of 40 ms [*t* = 3.069; *p* = 0.003] (Fig. 3b). Thus, pGFAP-BDNF mice presented larger paired-pulse facilitation than WT mice.

Given the different excitability of the hippocampal CA3–CA1 synapse in WT and pGFAP-BDNF mice, we checked the susceptibility of both groups of animals to a single, low-dose injection (8 mg/kg, i.p.) of kainate (Fig. 3c). Both WT (8 out of 18; 44.4%) and pGFAP-BDNF (8 out of 21; 38.1%) mice presented spontaneous hippocampal seizures after kainate injection, with no significant differences between groups (chi-square = 0.255; *p* = 0.613; Fig. 3f). In contrast, duration of spontaneous and train-evoked seizures in pGFAP-BDNF mice were longer than WT mice (Fig. 3d, e). The mean duration of kainate-evoked seizures was significantly different (*U* = 4.000, *p* = 0.003; Mann–Whitney rank-sum test) for both groups of mice (Fig. 3g). The presence of these longer-lasting seizures in pGFAP-BDNF mice (Fig. 3i) reduced the amplitude of fEPSPs evoked at the CA3–CA1 synapse in comparison with the effects evoked in WT animals (Fig. 3h). Overall, although these gain-of-BDNF-function experiments pointed out to a relevant role of the astrocytic BDNF in the modulation of the severity of TLE models, the use of pGFAP-BDNF is probably an artificial approach (e.g. the enhanced basal synaptic transmission could be due to local astrogliosis surrounding the electrodes in pGFAP-BDNF mice). Thereby, a loss-of-BDNF-function approach could reinforce our results and conclusions.

### BDNF from astrocytes regulates the hyperactivation of neuronal populations in an in vitro model of TLE

**Fig. 4:**
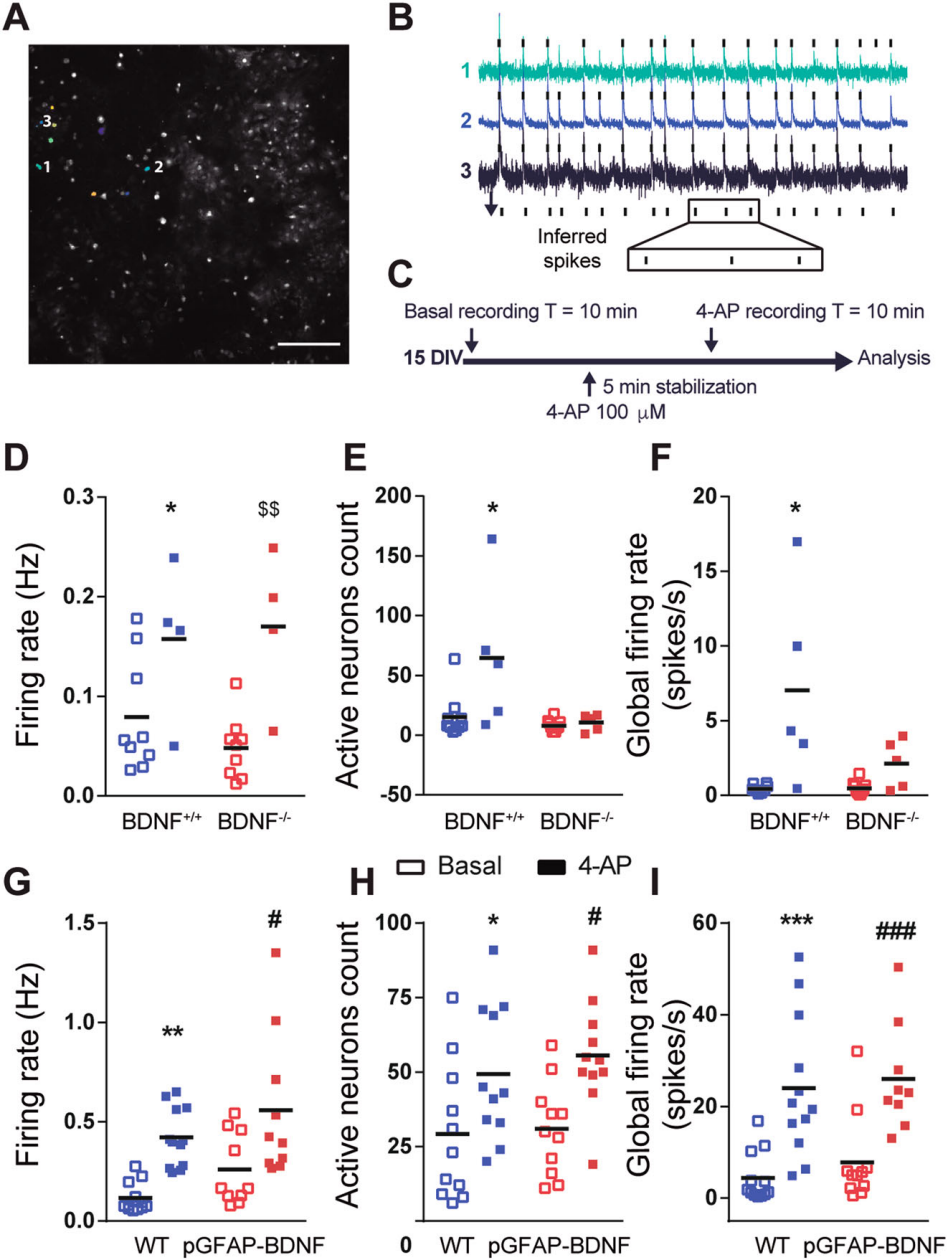
BDNF from astrocytes regulates neural network levels of activity in an in vitro model of temporal lobe epilepsy. **a** Representative average image of a hippocampal neurons-astrocytes co-culture at 15 DIV loaded with Fluo4-AM. **b** Calcium fluorescence traces from individual neurons highlighted in **a**. Black vertical lines indicate inferred spikes from the three neurons. **c** Experimental design: after 10 min recording in basal conditions, 4-AP is added to the culture and after 5 min of drug diffusion and stabilization, and neuronal activity is recorded for additional 10 min. **d** 4-AP treatment increased the firing rate of individual neurons in both conditions; cultured with BDNF^+/+^ or BDNF^−/−^ astrocytes (two-way ANOVA, 4-AP effect: *F*_(1,22)_ = 17.23, *p* < 0.001), indicating the effect of the treatment increasing neuronal activity. **e** 4-AP treatment has differential effect on the number of active neurons depending on if they were co-cultured with BDNF^+/+^ or BDNF^−/−^ astrocytes (two-way ANOVA, interaction effect: *F*_(1,26)_ = 5.204, *p* < 0.05; 4-AP effect: *F*_(1,26)_ = 6.419, *p* < 0.05); genotype effect: *F*_(1,26)_ = 8.881, *p* < 0.01). Post hoc analysis revealed a significant increase in the number of active neurons co-cultured with BDNF^+/+^ astrocytes after addition of 4-AP, but not in the ones co-cultured with BDNF^−/−^ astrocytes; after 4-AP treatment, the number of active neurons was different between BDNF^+/+^ and BDNF^−/−^ co-cultures. **f** 4-AP treatment increases the global firing rate in neurons in both conditions (two-way ANOVA, 4-AP effect: *F*_(1,26)_ = 7.266, *p* < 0.05); genotype effect: *F*_(1,26)_ = 5.654, *p* < 0.05). Post hoc analysis showed significance only in neurons co-cultured with BDNF^+/+^ astrocytes, but not in neurons co-cultured with BDNF^−/−^ astrocytes. Each point represents the average value of an individual experiment. Black lines show the mean of the group (*n* = 10 BDNF^+/+^ basal; 10 BDNF^−/−^ basal; 5 BDNF^+/+^ 4-AP; 5 BDNF^−/−^ 4-AP). **g** 4-AP treatment increased the firing rate in both conditions; individual neurons co-cultured with WT or pGFAP-BDNF astrocytes (two-way ANOVA, 4-AP effect: *F*_(1,40)_ = 22.17, *p* < 0.0001; genotype effect: *F*_(1,40)_ = 4.77, *p* < 0.05). **h** 4-AP treatment increased the number of active neurons in both conditions; in WT neurons co-cultured with WT or pGFAP-BDNF astrocytes (two-way ANOVA, 4-AP effect: *F*_(1,40)_ = 13.57, *p* < 0.001). **i** 4-AP treatment increased the global firing rate in both conditions; neurons co-cultured with WT or pGFAP-BDNF astrocytes (two-way ANOVA, 4-AP effect: *F*_(1,40)_ = 31.75, *p* < 0.0001). Each point represents the average value of an individual experiment. Black lines show the mean of the group (*n* = 12WT basal; 11pGFAP-BDNF basal; 11WT 4-AP; 11 BDNF^−/−^ 4-AP). Data were analyzed by two-way ANOVA followed by Bonferroni’s post hoc test. *p < 0.05, **p < 0.01, ***p < 0.001 compared to WT basal; ^$$^*p* < 0.001 compared to BDNF^−/−^ basal; ^#^*p* < 0.05, ^###^*p* < 0.001 compared to pGFAP-BDNF. Scale bar = 200 μm.

Previous studies have shown that in vitro and in vivo astrocytes produce and release BDNF^20–23^. To functionally explore the influence of astrocytic BDNF over the neuronal population response in an epileptic context we used a loss- and a gain-of-BDNF-function approach in vitro. We evaluated the activity of WT hippocampal neurons co-cultured with (i) BDNF^−/−^ or BDNF^+/+^ astrocytes and *(*ii) WT or pGFAP-BDNF astrocytes upon epileptic induction with 4-aminopyrimidine (4-AP)^26^. Neuronal activity was assessed by in vitro calcium imaging using Fluo4-AM as a fluorescence calcium indicator. Firing activity was inferred from each individual neuron and then averaged and considered as one individual experiment. First, the results showed that 4-AP induced an increase of the firing frequency in both BDNF^−/−^ and BDNF^+/+^ groups (Fig. 4d), indicating that loss of astrocytic BDNF does not interfere with the individual neuronal activity increase. In contrast, 4-AP treatment boosted a dramatic increase in the number of active neurons co-cultured with BDNF^+/+^ astrocytes, but not in the ones with BDNF^−/−^ astrocytes (Fig. 4e), indicating a recruitment of hyperactive neurons specifically in presence of BDNF^+/+^ astrocytes. We further evaluated the global levels of activity of the cultures by measuring the global firing rate, i.e. the average number of total spikes per unit time in the culture, and it showed an increase in BDNF^+/+^ cultures after the addition of 4-AP, but not in BDNF^−/−^ cultures (Fig. 4f).

Additionally, we repeated the experiment using WT or pGFAP-BDNF astrocytes. In summary, wild-type neurons co-cultured with pGFAP-BDNF astrocytes did not exhibited any difference compared with wild-type neurons co-cultured with WT astrocytes regarding to individual activity (Fig. 4g), the number of active neurons (Fig. 4h) and the global firing rate (Fig. 4i) upon 4-AP addition. An explanation to this lack of differences could be that 5 min stabilization of 4-AP treatment is not time enough to stimulate the conditional BDNF release from pGFAP-BDNF astrocytes as previously shown^20^. In conclusion, these results point to an important role of astrocytic BDNF in the recruitment of hyperactivated neurons during epileptic conditions.

### Normalization of TrkB.T1 levels in astrocytes or in neurons in the lithium-pilocarpine model

**Fig. 5:**
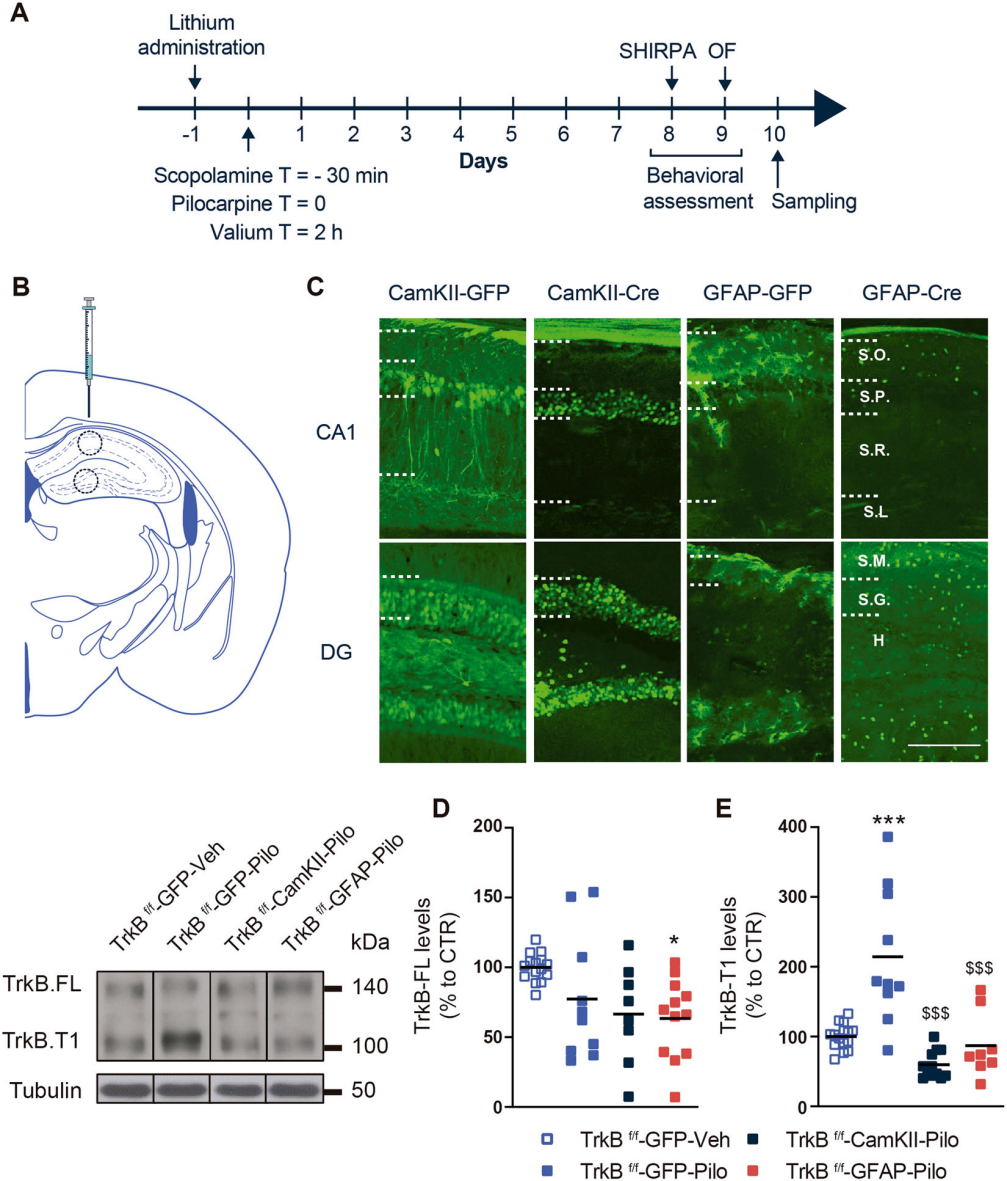
Effects of specific genetic deletion of TrkB in astrocytes or in neurons in the hippocampus of mice treated with lithium pilocarpine. Surgery was performed in mice in order to inject a specific AAV and after 3 weeks status epilepticus (SE) was induced using the lithium-pilocarpine model. For this, 24 h before pilocarpine application, lithium was administered. Thirty minutes before pilocarpine administration, the animals received scopolamine. Two hours after pilocarpine, the SE was stopped by using valium. Ten days after these procedures, the animals were sacrificed and analyzed (**a**). AAV-injection site in the mouse hippocampus: CA1 and DG (**b**). Representative images showing how CA1 and DG look after AAV injection are depicted (**c**). SO stratum orients, SP stratum pyramidale, SR stratum radiatum, SL-M stratum lacunosum moleculare, SM stratum moleculare, SG stratum granulare, H hilus. Scale bar: 200 μm. Representative immunoblots showing the levels of TrkB.FL and TrkB.T1 (**d**–**e**). The histograms represent the protein expression expressed as percentage of TrkB^f/f^-GFP-Veh. All data are shown as the mean ± SEM (*n* = 15 TrkB^f/f^-GFP-Veh; 10 TrkB^f/f^-GFP-Pilo; 8 TrkB^f/f^-GFAP-Cre-Pilo; 12 TrkB^f/f^-CaMKII-Cre-Pilo). Data were analyzed by one-way ANOVA followed by Tukey’s test. *p < 0.05, ***p < 0.001 compared to TrkB^f/f^-GFP-Veh. ^$$$^*p* < 0.001 compared to TrkB^f/f^-GFP-Pilo. ^#^*p* < 0.001 compared with TrkB^f/f^-CaMKII-Cre-Pilo.

Following the evaluation of the role of the BDNF produced by astrocytes in TLE models we next performed a second set of experiments aimed to evaluate the role of the astrocytic TrkB receptor in the lithium-pilocarpine model. To selectively eliminate TrkB expression from principal neurons of the CA1 and dentate gyrus (DG) or from astrocytes in the same hippocampal subregions, we used TrkB^f/f^mice. We injected these mice with adeno-associated viruses (AAVs) expressing Cre-recombinase under the control of CAMKII or GFAP promoter, leading to the deletion of TrkB expression in principal neurons or astrocytes, respectively. We performed bilateral hippocampal injections of AAV-CaMKII-GFP, AAV-GFAP-GFP, AAV-CaMKII-Cre-GFP, or AAV-GFAP-Cre-GFP (Fig. 5b). Next, the efficiency of the transduction in the TrkB^f/f^ mice was evaluated. We observed GFP-positive cells mostly in CA1 and DG. Neurons were mostly transduced in the *stratum pyramidale* of CA1. In contrast, GFP- and Cre-positive astrocytes were localized in the *stratum oriens*. In the DG, transduced neurons and astrocytes were mainly detected in the *stratum granulare* (Fig. 5c).

To determine whether SE affects TrkB receptor in our in vivo mouse model, animals that experienced SE for 2 h were sacrificed 10 days after pilocarpine administration and then analyzed (see scheme in Fig. 5a).TrkB protein levels were determined in the three conditions and compared with the control group (TrkB^f/f^-GFP-Veh) treated with vehicle. We did not detect significant changes in TrkB.FL expression either in the TrkB^f/f^-GFP-Pilo or in the TrkB^f/f^-CaMKII-Cre-Pilo groups (Fig. 5d). Only the TrkB^f/f^-GFAP-Cre-Pilo group displayed a significant reduction of TrkB.FL levels (Fig. 5d). This unexpected lack of TrkB.FL downregulation in the pilocarpine-treated control group could be due to detection limitations of the western blot approach. Thus, this result should be taken with caution since we cannot rule out a potential role of undetectable downregulated TrkB.FL levels. Conversely, a significant and consistent increase in the truncated form of TrkB (TrkB.T1) was found in the control animals that displayed SE (TrkB^f/f^-GFP-Pilo) (Fig. 5e). These results are in line with previous studies^27^ and demonstrated that SE in our in vivo model induced a consistent TrkB.T1 increase. Interestingly, the TrkB.T1 increase was prevented in TrkB^f/f^-CaMKII-Cre-Pilo and TrkB^f/f^-GFAP-Cre-Pilo groups suggesting that neurons or astrocytes that would increase TrkB.T1 levels were efficiently targeted with the AAV expressing Cre-recombinase strategy.

### Normalization of TrkB.T1 levels in astrocytes prevents hyperlocomotion and spatial learning deficits induced by pilocarpine

**Fig. 6:**
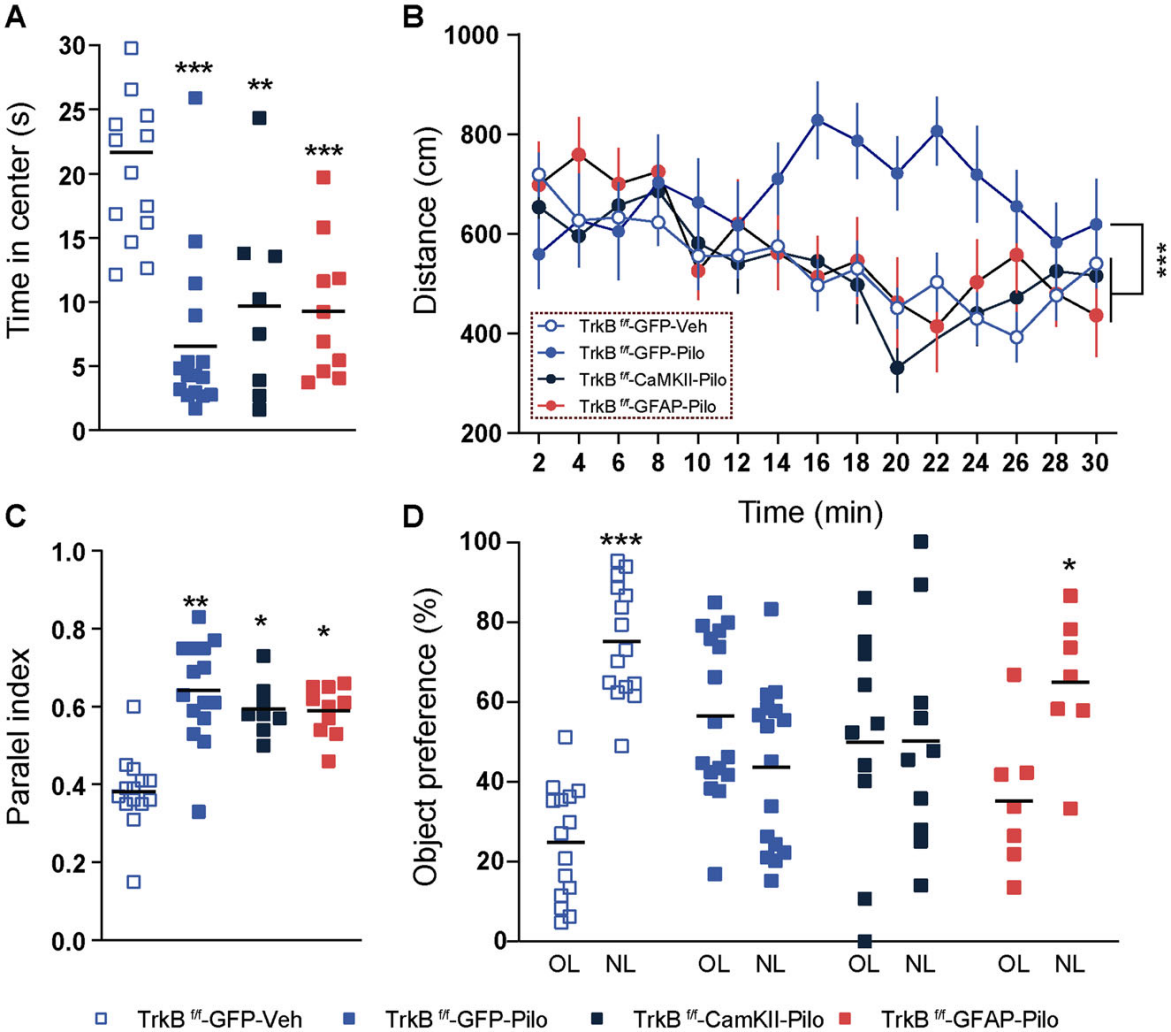
Lithium pilocarpine induced behavioral effects in mice with genetic deletion of TrkB in neurons or in astrocytes. TrkB^f/f^-GFP-Veh, TrkB^f/f^-GFP-Pilo, TrkB^f/f^-GFAP-Cre-Pilo, and TrkB^f/f^-CaMKII-Cre-Pilo mice were subjected to an open field (day 7 after pilocarpine treatment) and to a novel object location test (NOL, day 9 after pilocarpine treatment) assays. In the open field, **a** time spent in the center of the arena, **b** locomotor activity, and **c** parallel index were monitored for 30 min in all groups of mice. **a** Locomotor activity measurement indicated significant differences between groups (two-way ANOVA, group effect: *F*_(3,675)_ = 15.78, *p* < 0.001), post hoc analysis indicated that TrkB^f/f^-GFP-Pilo mice were the only ones who were significantly different compared with TrkB^f/f^-GFP-Veh mice (*p* < 0.001). **b** In the variable time spent in the center of the arena we identified a general significant effect from all groups treated with pilocarpine compared with TrkB^f/f^-GFP-Veh mice (one-way ANOVA: *F*_(3,44)_ = 13.25, *p* < 0.001). **c** In the variable parallel index, we identified a general significant effect from all groups treated with pilocarpine compared with TrkB^f/f^-GFP-Veh mice (one-way ANOVA: *F*_(3,44)_ = 6.27, *p* < 0.001). **d** In the NOL test, the percentage of time exploring the displaced object (new location, NL, 24 h after first exposure) and the unmoved object (old location, OL) was compared. Two-way ANOVA indicated a significant interaction effect groups × object location (*F*_(3,88)_ = 14.46, *p* < 0.001) and post hoc analysis indicated that only TrkB^f/f^-GFP-Veh (*p* < 0.001) and TrkB^f/f^-GFAP-Cre-Pilo (*p* < 0.05) mice showed significant preference for the displaced object. Bars represent mean ± SEM. Data were analyzed by two-way analysis (**a**, **d**) or one-way (**b**, **c**) of variance (ANOVA) with Bonferroni’s test as a post hoc. *p < 0.05 and ***p < 0.001 compared with vehicle controls. TrkB^f/f^-GFP-Veh (*n* = 15), TrkB^f/f^-GFP-Pilo (*n* = 16), TrkB^f/f^-GFAP-Cre-Pilo (*n* = 8), and TrkB^f/f^-CaMKII-Cre-Pilo (*n* = 10).

Seven days after pilocarpine treatment we performed a behavioral characterization of TrkB^f/f^ mice injected with AAV-GFP and treated with vehicle and TrkB^f/f^ mice injected with AAV-GFP or with AAV-GFAP-Cre or with AAV-CaMKII-Cre and treated with pilocarpine (TrkB^f/f^-GFP-Veh, TrkB^f/f^-GFP-Pilo, TrkB^f/f^-GFAP-Cre-Pilo, TrkB^f/f^-CaMKII-Cre-Pilo groups, respectively). On day 7, we subjected the mice to the open field paradigm. Statistical analysis showed no significant differences between the groups TrkB^f/f^-GFAP-Cre-Pilo, TrkB^f/f^-CaMKII-Cre-Pilo, and TrkB^f/f^-GFP-Veh in spontaneous locomotor activity (Fig. 6b). In contrast, TrkB^f/f^-GFP-Pilo mice displayed a significant increase on locomotor activity respect to the TrkB^f/f^-GFP-Veh mice (Fig. 6b). This result indicated a strong effect on locomotor activity mediated by the pilocarpine treatment in TrkB^f/f^-GFP-Pilo but not in TrkB^f/f^-GFAP-Cre-Pilo and TrkB^f/f^-CaMKII-Cre-Pilo mice. We also evaluated time spent in the center of the arena as a measure of anxiety-like behavior. Results showed a significant decrease of this parameter in the TrkB^f/f^-GFP-Pilo and TrkB^f/f^-GFAP-Cre-Pilo and TrkB^f/f^-CaMKII-Cre-Pilo groups compared to TrkB^f/f^-GFP-Veh mice (Fig. 6a). This decrease suggested a general and strong effect induced by pilocarpine in anxiety-like measurements. We also evaluated the parallel index (1.0 means walking straight) as a measure of spatial navigation strategy in mice^28^. We observed that TrkB^f/f^-GFP-Pilo and TrkB^f/f^-GFAP-Cre-Pilo and TrkB^f/f^-CaMKII-Cre-Pilo groups displayed a significant increase on parallel index compared to TrkB^f/f^-GFP-Veh mice (Fig. 6c). This increase indicated that pilocarpine-treated mice walked aberrantly straight in contrast to normal spatial navigation strategies observed in healthy mice. Finally, we subjected all the groups to the novel object location (NOL) test on day 9 after the pilocarpine treatment. In the NOL test, 24 h after the training phase, TrkB^f/f^-GFP-Veh mice spent more time exploring the object placed at a new location (Fig. 6d). In contrast, TrkB^f/f^-GFP-Pilo and TrkB^f/f^-CaMKII-Cre-Pilo mice did not display any preference for either object (Fig. 6d). Interestingly, TrkB^f/f^-GFAP-Cre-Pilo mice spent more time exploring the object placed at a new location (Fig. 6d). These results revealed spatial memory deficits in both TrkB^f/f^-GFP-Pilo and TrkB^f/f^-CaMKII-Cre-Pilo mice but not in TrkB^f/f^-GFAP-Cre-Pilo mice compared to TrkB^f/f^-GFP-Veh mice.

### Normalization of TrkB.T1 levels in astrocytes or in neurons are both neuroprotective in the lithium-pilocarpine model

**Fig. 7:**
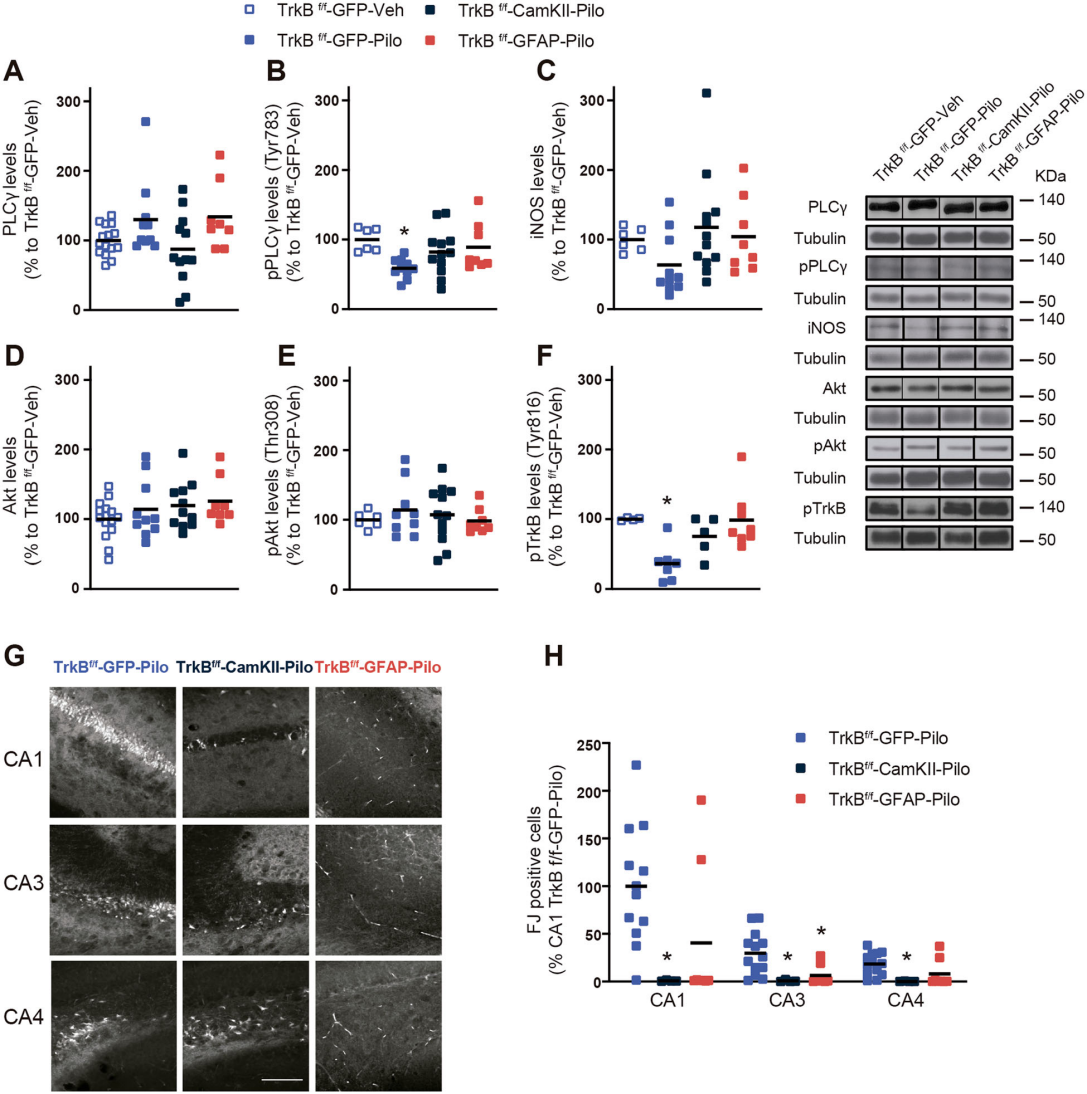
Pilocarpine-induced biochemical effects in the hippocampus of mice with genetic deletion of TrkB in neurons or in astrocytes. Protein levels of the principal TrkB downstream signaling proteins including **a** PLCγ, **b** pPLCγ, **c** Akt, and **d** pAkt and iNOS Histograms represent the protein expression expressed as the percentage of TrkB^f/f^-GFP-Veh. Representative immunoblots are shown. All data are shown as the mean ± SEM (TrkB^f/f^-GFP-Veh (*n* = 15); TrkB^f/f^-GFP-Pilo (*n* = 10); TrkB^f/f^-GFAP-Cre-Pilo (*n* = 8); TrkB^f/f^-CaMKII-Cre-Pilo (*n* = 12)). Data were analyzed by one-way ANOVA followed by Tukey’s test. *p < 0.05 compared with TrkB^f/f^-GFP-veh. **g** Representative images showing Fluoro-Jade-positive cells (FJ+) in the three experimental conditions in CA1, CA3, and CA4. Scale bar: 100 μm. The histogram represents the number of FJ+ cells expressed as the percentage of the number of positive cells found in the CA1 of TrkB^f/f^-GFP-Pilo. All data are shown as mean ± SEM (TrkB^f/f^-GFP-Pilo (*n* = 13); TrkB^f/f^-CaMKII-Cre-Pilo (*n* = 4); TrkB^f/f^-GFAP-Cre-Pilo (*n* = 8)). Data were analyzed by one-way ANOVA followed by Tukey’s test. *p < 0.05 compared with TrkB^f/f^-GFP-Pilo.

To infer the possible roles of the downstream TrkB pathway in vivo after pilocarpine induced neuronal injury, key proteins of the TrkB signaling pathway were evaluated 10 days after SE. First, PLCγ levels failed to show significant differences between groups (Fig. 7a). In contrast, the phosphorylated form of PLCγ (pPLCγ) was significantly decreased in the TrkB^f/f^-GFP-Pilo mice. This decrease was not observed in both groups that have either neurons or astrocytes depleted of TrkB expression (Fig. 7b). Regarding the levels of the inducible form of NOS (iNOS), a tendency towards a decreased expression was found in TrkB^f/f^-GFP-Pilo mice. Both TrkB^f/f^-CaMKII-Cre-Pilo and TrkB^f/f^-GFAP-Cre-Pilo groups did not present significant changes in this protein (Fig. 7c). Akt and phospho-Akt protein levels were not altered in any of the groups of our study (Fig. 7d, e). Finally, we observed that TrkB phosphorylation levels at the tyrosine 817 were reduced in the hippocampus of the TrkB^f/f^-GFP-Pilo mice, but completely unaffected in the hippocampus from both, TrkB^f/f^-CaMKII-Cre-Pilo and TrkB^f/f^-GFAP-Cre-Pilo mice (Fig. 7f). To evaluate the consequences of TrkB genetic deletion in neurons or astrocytes in neuroprotection, we performed Fluoro-Jade (FJ) staining in hippocampal sections from mice euthanized 10 days after SE in three different conditions: TrkB^f/f^-GFP-Pilo, TrkB^f/f^-CaMKII-Cre-Pilo, and TrkB^f/f^-GFAP-Cre-Pilo. SE preferentially causes death in CA1 pyramidal cells as evidenced by FJ staining in sections from TrkB^f/f^-GFP-Pilo mice (Fig. 7g). CA3 and CA4 hippocampal regions showed less cell death. To evaluate whether TrkB.T1 normalization involve changes in neuronal death after SE, we compared the results from the TrkB^f/f^-GFP-Pilo group with the TrkB^f/f^-CaMKII-Cre-Pilo and TrkB^f/f^-GFAP-Cre-Pilo groups. A general neuroprotective effect in all the hippocampal regions (CA1, CA3, and DG) was observed in both TrkB^f/f^-CaMKII-Cre-Pilo and TrkB^f/f^-GFAP-Cre-Pilo compared to TrkB^f/f^-GFP-Pilo (Fig. 7g, h).

## Discussion

It has been shown that both BDNF and TrkB are required for epileptogenesis^29,30^, suggesting that TrkB and its downstream signaling pathways are attractive therapeutic targets for epilepsy^31^. Moreover, BDNF and TrkB downstream signaling plays an important role in neuronal function^32^ and deficiency of this pathway has been linked to important brain diseases, memory loss and cognitive alterations^33^. Therefore, the therapeutic potential of targeting the BDNF or TrkB molecules in TLE has remained in an impasse due to off-target effects. The present work tackles this challenge and enables a novel approach in the manipulation of the promising BDNF and TrkB molecules in astrocytes to treat TLE.

Here we prove that BDNF from astrocytes increases neuronal activation and toxicity in pathological conditions supporting previous literature showing astrocytic control of neuronal activity and synaptic currents^34–38^. Together, these data point out to an important function of astrocytic BDNF in the neuronal communication. Concretely, our results point out to a possible major role of astrocytic pro-BDNF upregulation in the modulation of neurodegenerative processes induced by our TLE models as previously demonstrated^39,40^, whereas more modest changes in mBDNF dynamics could account for the changes observed on enhanced synaptic transmission in the pGFAP-BDNF model and the increase of firing neurons in the in vitro 4-AP model as previously demonstrated for this isoform^41,42^.

TrkB receptor and its truncated isoform TrkB.T1 are expressed in astrocytes and neurons and could mediate the regulation of neuronal hyperactivation in the first stages of TLE^31,43,44^. The present results demonstrated that mostly pro-BDNF (although a role from mBDNF cannot be ruled out) may have a key role in the development of SE, and that a reduction of TrkB.T1 (we cannot rule out an effect from undetected TrkB.FL downregulation) expression specifically in astrocytes is neuroprotective and rescues the performance in the NOL task. This is important since TrkB manipulation can alter synaptic plasticity and hippocampal-dependent learning^45–47^.

The role of BDNF and TrkB during different phases of TLE is complex. Previous work showed therapeutic effects of long-term BDNF supplementation in the epileptic hippocampus by reducing degenerating neurons^48^. Compared to our results, this differential effect can be explained by the different temporal approach of the BDNF release. Thus, an acute and sustained increase on BDNF levels during the first stages of TLE can induce an aberrant neuronal hyperexcitability and death while a late-onset and chronic treatment with this neurotrophin induces synaptic changes and promotes neuronal survival^49^.

Importantly, we found here an outstanding increase in the expression of TrkB.T1 as predicted^27,50^. Furthermore, TrkB.T1 was diminished/normalized in both groups of mice that have TrkB expression genetically disrupted in neurons or in astrocytes, validating the neuroprotective effect mediated by a reduction of TrkB.T1 expression after SE in a cell-specific manner. Upon excitotoxic conditions, a change in the TrkB isoforms occurs, decreasing the levels of TrkB.FL and increased TrkB.T1^51^. The functional impact of TrkB.T1 increase has been controversial^16,51^. According to our results, normalization of TrkB.T1 levels might underlie the neuroprotective effect observed upon TrkB expression reduction, supporting the hypothesis of deleterious effect of TrkB.T1 increase in the epileptic context.

TrkB-dependent signaling has been shown to be downregulated in excitotoxic models^51^. Indeed, we observe a decrease in the phosphorylation of PLCγ upon pilocarpine treatment, which is a main affected molecular pathway by which SE-induced TrkB activation promotes its epileptogenic consequences^52^. Moreover, conditional reduction of TrkB expression in both neurons and astrocytes is able to rescue the decrease of both pPLCγ and pTrkB, probably due to the reduction of TrkB.T1 that hampers the activation of TrkB.FL receptor^13,15,53^. Conversely, pilocarpine treatment did not modify the BDNF-dependent phosphorylation of Akt as previously described^51^.Therefore, our results reveal for the first time a major participation of astrocytic TrkB in the cell death and cognitive deficits related to TLE models.

Additionally, we have observed some effects probably due to a delayed time point in which we evaluated the mice. First, despite the weak neuronal death observed in mice genetically depleted of TrkB.T1 and TrkB.FL and treated with pilocarpine, we cannot discard that some neuronal death occurred during the first stages after pilocarpine treatment and we missed it out. Second, the presence of degenerating neurons in some hippocampal subregions (CA1 and CA3) but not in others (CA4) suggests differential sensitivity to pilocarpine of neurons from different hippocampal sub-fields. Thus, it is possible that these different degeneration dynamics have been neglected by focusing our experiments only in one time point. Third, although we observed neuroprotection in different hippocampal sub-fields, we did not detect changes in phospho-Akt, a well-known pathway involved in neuronal survival^54^. We cannot exclude that changes in phospho-Akt, among others, could have taken place during the first hours just after the pilocarpine treatment. Overall, our results point to astrocytic BDNF and TrkB as promising therapeutic targets for the treatment of TLE.

## Materials and methods

### Animals

For in vivo experiments in 10-week-old adult male mice (all in a C57BL/6 strain background) we used pGFAP-BDNF mice that express BDNF under the GFAP promoter^20^. For TrkB deletion, TrkBf/f mice were generated in which the NTRK2 exon S was flanked with LoxP sequences^55^. We used P1–P3 BDNF^+/+^ and BDNF^−/−^ mice^56^ (IMSR Cat# EM:00247, RRID:IMSR_EM:00247) to obtain astrocytes in vitro devoid of BDNF. Mouse genotyping for pGFAP-BDNF, TrkB^f/f^, and BDNF^−/−^ mice was carried out from a tail biopsy as previously described^57^. The animals were housed with access to food and water ad libitum in a colony room kept at 19–22 °C and 40–60% humidity, under a 12:12 h light/dark cycle. Age of the mice was specified in each figure legend according to the experimental approach requirements. Experimental animals (all male) were used in accordance with the ethical guidelines (Declaration of Helsinki and NIH, publication no. 85-23, revised 1985, European Community Guidelines, and approved by the local ethical committee (University of Barcelona, 225/17 and Generalitat de Catalunya, 404/18).

### Viral constructs and stereotaxic injection

Following anesthesia with ketamine/xylazine (100 and 10 mg/kg, respectively), we performed bilateral hippocampal injections of rAAV5-CaMKII-GFP (AV4587C, 5.3 × 10^12^ GCs), rAAV5-GFAP-GFP (AV4658b, 9.1 × 10^12^ GCs), rAAV5-CaMKII-Cre-GFP (AV6450B, 3.5 × 10^12^ GCs), or rAAV5-GFAP-Cre-GFP (AV6454B, 4.8 × 10^12^ GCs). All the AAVs were purchased to the Virus Vector Core UNC (G44 Wilson Hall, CB 7352, Chapel Hill, NC. 27599-7352). We used the following coordinates (millimeters) from bregma (anteroposterior and lateral) and from skull (dorsoventral); anteroposterior: −2.0; Lateral +/−1.25, and dorsoventral: −1.3 (CA1) and −2.1 (GD). The cannula was left to deliver 1 μl of 1:1 virus in each depth during 2 min and five additional minutes were left to have complete virus diffusion. After 2 h of careful monitoring, mice were returned to their home cage for 3 weeks before starting the lithium-pilocarpine model and subsequent behavioral and biochemical analysis.

### Pilocarpine treatment

Animals were subjected to the lithium-pilocarpine model of TLE. Briefly, mice were treated intraperitoneally with lithium (LiCl, 423 mg/kg, i.p.) 20–23 h prior to administration of scopolamine, an anti-muscarinic molecule (1 mg/kg, i.p.). Thirty minutes after the administration with methyl-scopolamine, animals were treated with pilocarpine (45 mg/kg, i.p.). Control animals were injected with saline solution. *Status epilepticus* (SE) was stopped after approximately 120 min with an intraperitoneal injection of valium (10 mg/kg, i.p.). Only animals that displayed 120 min of SE survived and showed the following levels of epileptic seizure were selected: mouth and facial movement, tremors, head nodding, and forelimb clonus.

### Electrophysiological experiments

Male, 8-month-old pGFAP-BDNF mice and their littermate wild-type (WT) controls of the same age and sex were prepared for the in vivo electrophysiological study of hippocampal functions. These experiments were carried out in the animal facilities of the Pablo de Olavide University. Upon their arrival animals were housed with access to food and water ad libitum and kept at 21 °C and 50% humidity, under a 12:12 h light/dark cycle. Experiments were carried out following European Union Council (2010/276:33–79/EU) guidelines and Spanish (BOE 34:11370-421, 2013) regulations for the use of laboratory animals in chronic experiments. Experiments were also approved by the local Ethics Committee of Pablo de Olavide University (document no 06/03/2018/025).

As already described^21^, animals were anesthetized with 1.2% isoflurane (Astra Zeneca, Madrid, Spain) delivered via a mask adapted to mouse head (Cibertec, Madrid, Spain). Then, animals were implanted with a bipolar stimulating electrode at the right Schaffer collateral-commissural pathway of the dorsal hippocampus (1.5 mm posterior to bregma; 2 mm lateral; 1.3 mm from brain surface^58^) and with two recording electrodes at the ipsilateral *stratum radiatum* underneath the CA1 area (2.2 mm posterior to bregma; 1.2 mm lateral; 1.0–1.5 mm from brain surface). Electrodes were made of 50 μm, Teflon-coated tungsten wire (Advent Research Materials, Eynsham, UK). The final location of the recording electrodes was determined following the field potential depth profile evoked by paired (40 ms of interval) pulses presented to the stimulating electrode^59^. Two bare silver wires were affixed to the skull as ground. Electrodes were connected to a 6-pin socket (RS-Amidata, Madrid, Spain) that was affixed with dental cement to the cranial bone. After surgery, animals were kept in individual cages provided with food and water ad libitum until the end of the experimental sessions.

To allow a proper recovery of the experimental animals, recording sessions were started 1 week after surgery. For input/output curves (Fig. 1a), monosynaptic fEPSP were evoked in the CA1 area by single (100 µs, square, and negative–positive) pulses applied to Schaffer collaterals. A total of 20 pulses were presented at increasing intensities, from 20 to 400 µA, in steps of 20 µA. In order to avoid unwanted interactions between stimuli, intervals of 30 s were allowed between successive pulses^60^.

For the paired-pulse facilitation at the CA3–CA1 synapse (Fig. 1b), we used the above described pulses, but presented in pairs at increasing inter-pulse intervals (10, 20, 40, 100, 200, and 500 ms). Stimulus intensities were set at 30–40% of the intensity necessary for evoking a maximum fEPSP response^59,61^. Intervals between pair of pulses were set at 30 s to avoid unwanted interactions evoked by pre- or postsynaptic mechanisms.

### Kainate administration and recording of evoked seizures

In order to determine the propensity of WT and pGFAP-BDNF mice to generate convulsive seizures, animals were injected (i.p.) with the α-amino-3-hydroxy-5-methyl-4-isoxazolepropionic acid (AMPA)/kainate receptor agonist kainic acid (8 mg/kg; Sigma, Saint Louis, Missouri, USA) dissolved in 0,1 M phosphate-buffered saline (PBS) pH = 7.4 as previously described to increase hippocampal excitability in vivo^62,63^. As illustrated in Fig. 1c, local field potentials were recorded in the CA1 area from 10 min before to 35 min after the kainate injection. Before kainate administration, animals were presented with several pair of stimuli (40 ms of inter-pulse interval); these paired pulses were repeated in each animal right after the end of each spontaneous seizure. Injected animals were presented with a stimulus session (five 200 Hz, 100 ms trains of pulses at a rate of 1/s) 5 min after kainate administration.

### Astrocyte/neurons co-cultures

Primary astrocyte cultures were obtained from P1 to P3 BDNF^+/+^ and BDNF^−/−^ mouse pups^56^ (IMSR Cat# EM:00247, RRID:IMSR_EM:00247), and from P1 to P3 WT and pGFAP-BDNF mouse pups^20^ by hippocampal dissections. Extracted tissue was dissociated and placed in 25 cm^2^ flasks in an MEM 1Px conditioned media NM-15 (20% fetal bovine serum; Gibco-BRL, Renfrewshire, Scotland, UK; d-Glucose 90 mM; Buchs, Switzerland) with l-glutamine and Earle’s salts (Gibco-BRL) and placed in an incubator at 37 °C with 5% CO_2_. A tail biopsy was obtained from each pup for genotyping. After two passages cultures were purified by agitating in a shaker during 10 min at 400 r.p.m. Medium with undesired floating cells was replaced and flasks were placed in an incubator for 2 h at 37 °C. Next, flasks were agitated again for 16–18 h at 250 r.p.m. Finally, medium with floating cells was replaced with new medium. Once astrocytes reached confluence, they were seeded in 24-well plaques and allowed to reach confluence. Then, one day before the addition of hippocampal neurons, astrocyte cultures were pre-incubated with neurobasal medium (GIBCO, Renfrewshire, Scotland, UK) containing 1 ml per 50 ml of B27 supplement (Gibco-BRL) and 500 μl of GlutaMAX (100×) (Gibco-BRL). Hippocampal neurons were prepared from E18 B6CBA mouse embryos (pregnant mice from Charles River, Saint Germain Nuelles, France). The neuronal cell suspension was low density seeded in the 24-well plaques already containing astrocyte monolayer (10,000 cells cm^2^).

### Calcium imaging recording and analysis

Calcium fluorescence recordings were carried out at 15 DIV using cell-permeant calcium sensitive dye Fluo4-Acetoxymethyl ester (Fluo4-AM) (Invitrogen, Thermo Fisher, Waltham, MA, USA). Cultures were incubated for 20 min at 37 °C in their own Neurobasal medium in the presence of 1 µM Fluo4. After incubation, cultures were placed in a 35 mm diameter glass bottom chamber (P35G-0-14-C; MatTek Corporation) for recording. Recordings were carried out in pH-stable (7.4) external medium (EM). EM consisted of: HEPES (4-(2-hydroxyethyl)-1-piperazineethanesulfonic acid), 10 mM; NaCl, 128 mM; KCl, 4 mM; glucose, 10 mM; sucrose, 45 mM; CaCl_2_, 2 mM; MgCl_2_, 1 mM. The recording chamber was mounted on an Olympus IX70 inverted microscope equipped with a Hamamatsu Orca Flash 4.0 V2 (Digital CMOS camera C11440-22CU) camera and Dual OptoLED power supply (Cairn Research Ltd) as a source of light. Fluorescence images were acquired at room temperature (RT) with a ×10 objective (20 frames/s). Neurons were recorded during 10 min for stabilization, afterward for induction of epileptiform activity, 100 μM 4-aminopirimidine or vehicle was added to the EM. After 5 min allowing the diffusion of the drug, activity was recorded during additional 10 min.

Analysis of calcium imaging recordings was performed with NETCAL^64^, a custom-made software package developed in Matlab (MathWorks). Recordings were first time-averaged across the whole recording to obtain a clear picture of the cell bodies. Cells were automatically detected as regions of interest (ROIs) using the time-averaged image. Traces were acquired for each ROI by spatially averaging between its pixels across the whole recording. Each trace was smoothed, and its baseline corrected. To estimate the baseline, a reference point was taken for each 50 s block, and its position within the block determined by the value closest to the 10% lower fluorescence percentile within that block. A smoothing spline was then fitted using this point set. This baseline was subtracted from the original trace and finally normalized to 100 × (*F* − *F*_0_)/*F*_0_ relative units.

Using a supervised machine learning approach, neuronal activity traces were selected. First, traces were visually examined and a subset belonging to neuronal activity class was manually selected. Then the classifier was trained with this subset using adaptive boosting (AdaBoost), machine learning meta-algorithm. The resulting classification was visually inspected and refined by changing the training set as required.

Spikes were inferred using a modified Peeling method^65^. For the underlying calcium model, a single decaying exponential with non-saturating dynamics with amplitude 1% Δ*F*_0_/*F*_0_ and decay 3 s was used on all experiments. Active neurons were considered when at least one spike was detected during the recording.

### Western blot

Animals were euthanized by cervical dislocation. The two hippocampi were dissected out, frozen using CO_2_ pellets, and stored at −80 °C until use. Briefly, the tissue was lysed by sonication in 150 ml of lysis buffer (PBS, 10 μg/ml Nonidet P-40, 1 mM PMSF, 10 μg/ml aprotinin, 1 μg/ml leupeptin, and 2 mM sodium orthovanadate). After lysis, samples were centrifuged at 12,000 r.p.m. for 15 min. Supernatant proteins (15 mg) from total brain regions extracts were loaded in polyacrylamide gels (SDS–PAGE) at different polyacrylamide concentrations and transferred to nitrocellulose membranes (Whatman Schleicher & Schuell, Dassel, Germany) during 1 h. Membranes were blocked in TBS-T solution (150 mM NaCl, 20 mM Tris-HCl, 0.1% Tween 20, pH 7.5) plus 5% non-fat dry milk and 5% bovine serum albumin. Membranes were probed with the following antibodies: anti-BDNF rabbit 1:1000 (ICOSAGEN, #327-100, clone 3C11), anti-GFAP rabbit 1:1000 (Agilent Cat# Z0334, RRID:AB_10013382), anti-TrkB mouse 1:1000 (BD Biosciences Cat# 610101, RRID:AB_397507), anti-PLC1γ rabbit 1:1000 (Technology Cat# 2822, RRID:AB_2163702), anti-pPLC1γ (Tyr783) rabbit 1:1000 (Cell Signaling Technology Cat# 2821, RRID:AB_330855), anti-Akt rabbit 1:2000 (Cell Signaling Technology Cat# 9272, RRID:AB_329827), anti-pAkt (Thr308) rabbit 1:1000 (Cell Signaling Technology Cat# 9275, RRID:AB_329828), anti-pTrkB (Tyr816) rabbit 1:1000 (bcam Cat# ab75173, RRID:AB_1281172), and anti-iNOS rabbit 1:1000 (Novus Cat# NB300-605, RRID:AB_10002794). All membranes were incubated with the primary antibodies overnight at 4 °C by shaking in PBS with 0.02% sodium azide. After washed with TBS-T, membranes were incubated with anti-rabbit or anti-mouse horseradish peroxidase-conjugated secondary antibodies (1:2000; Promega). Secondary antibody binding was detected by the enhanced chemiluminescence substrate kit (Santa Cruz Biotechnology, Santa Cruz, Ca, USA). For loading control a mouse monoclonal antibody for α-tubulin was used (Sigma-Aldrich Cat# T9026, RRID:AB_477593). ImageJ software was used to quantify the different densitometry immunoreactive bands relative to the intensity of the α-tubulin in the same membranes. Data are expressed as the mean ± SEM of band density.

### Tissue fixation and immunofluorescence

Mice were euthanized by cervical dislocation. Left hemispheres were removed and fixed for 72 h in 4% paraformaldehyde (PFA) in PBS. Forty-micrometer coronal sections were obtained using a Leica vibratome (Leica VT1000S). Next, free floating sections were washed three times in PBS, treated with NHCl for 30 min, and washed again three times with PBS. Floating sections were permeabilized and blocked with PBS containing 3% Triton X-100, 0.02% Azide, 2% BSA, and 3% NGS (Ab buffer) for 1 h at RT. After three washes in PBS, brain slices were incubated overnight at 4 °C with FITC-conjugated GFP primary antibody (AB6662; 1:500, Abcam) for signal enhancement. Sections were washed three times and incubated for 2 h at RT with fluorescent secondary antibody Alexa Fluor 488 goat anti-mouse (1:400; from Jackson Immuno Research, West Grove, PA, USA). Sections were analyzed using a two-photon confocal microscope (Leica SP5).

### Fluoro-Jade staining and counting

To assess degenerating neurons in the hippocampus, mice were sacrificed 10 days after the administration of pilocarpine or vehicle. Left hemispheres were removed and kept in PFA during 3 days with constant shaking. At this point, PFA was replaced for PBS until the brains were cut. Forty-micrometer coronal sections were obtained using the Leica vibratome (Leica VT1000S). Brain sections were mounted with distilled water onto SuperFrost slides (Thermo Scientific™ SuperFrost™ Microscope Slides) and dried at RT. The tissue was fully dry within 30 min at which time it was immersed in 100% ethyl alcohol for 3 min followed by a 1 min change in 70% alcohol and a 1 min change in distilled water. The slides were then transferred to a solution of 0.06% potassium permanganate for 15 min and were gently shaken on a rotating platform. The slides were rinsed for 1 min in distilled water and were then transferred to the Fluoro-Jade staining solution where they are gently agitated for 30 min. A 0.01% stock solution of the dye was prepared by dissolving 10 mg Fluoro-Jade in 100 ml of distilled water. The 0.001% working solution of Fluoro-Jade was prepared by adding 10 ml of the stock Fluoro-Jade solution to 90 ml of 0.1% acetic acid in distilled water. After staining, the sections were rinsed with three 1 min changes of distilled water. Excess water was drained off, and the slides were rapidly air dried. When dry, the slides were immersed in xylene and then mounted with Mowiol mounting media.

### Confocal and epifluorescence imaging and analysis

Sections were analyzed using an Olympus BX60 epifluorescent microscope equipped with an Orca-ER cooled CDD camera (Hamamatsu Photonics, Hamamatsu, Japan) with the Hokawo (Hamamatsu) image software acquisition. Three hippocampal sections were analyzed per animal and for each section five images were taken using the ×20 objective. To quantify the degenerating cells, the mean area of CA1, CA3, and CA4 was measured for each section and the number of Fluoro-Jade C (FJC)-positive cells inside this area was counted using FIJI software (Wayne Rasband, NIH). The mean value of all animals from each group was obtained and represent as % related to the control group and as % related to the CA1 of the control group.

### Behavioral tests

To provide detailed mid-term general phenotype assessment 7–9 days after the pilocarpine treatment, mice were tested using the phenotype assessment protocol called SHIRPA, the OF, and the NOL test, all these tests have been used to detect mid-term behavioral alterations such as general neurological alterations, hyperlocomotion activity, and memory impairments induced by pilocarpine^66–71^. First, materials for the SHIRPA evaluation consisted of a transparent Perspex viewing jar, a clean cage, a wire grid cage lid, and a stopwatch. During the procedure, each mouse was removed from the home cage, evaluated individually in the viewing jar, and then placed into the clean cage for further evaluation. Finally, animals were placed on the grid cage and last items were evaluated. The viewing jar and the testing cage were cleaned with 70° ethanol between animals to avoid the possibility of olfactory cues affecting behavior. The data from individual components of the modified SHIRPA were pulled to provide a general SHIRPA index as previously described^72^. For further information about the SHIRPA procedure, more detailed information is provided in the Mouse Phenome Database at the Jackson Laboratory’s website: https://phenome.jax.org/projects/Jaxpheno7/protocol#SHIRPA.

To check spontaneous locomotor activity, anxiety-like parameters, and navigation we used the OF. Briefly, the apparatus consisted of a white square arena measuring 40 × 40 × 40 cm in length, width, and height, respectively. Dim light intensity was 60 lux throughout the arena. Animals were placed on the arena center and allowed to explore freely for 30 min. Spontaneous activity was measured. At the end of each trial, any defecation was removed, and the apparatus was wiped with 30% ethanol. Animals were tracked and recorded with SMART junior software (Panlab, Spain).

The NOL memory task evaluates spatial memory and is based on the ability of mice to recognize when a familiar object has been relocated. Exploration took place in an open-top arena with quadrangular form (45 × 45 cm). The light intensity was 40 lux throughout the arena. Mice were first habituated to the arena in the absence of objects (1 day, 30 min). Some distal cues were placed throughout the procedure. On the third day during the acquisition phase mice were allowed to explore two duplicate objects (A1 and A2), which were placed close to the far corners of the arena for 10 min. After a delay of 24 h, one object was placed in the corner diagonally opposite. Thus, both objects in the phase were equally familiar, but one was in a new location. The position of the new object was counterbalanced between mice. Animals were tracked and recorded with SMART Junior software.

### Experimental design and statistical analysis

Sample sizes were determined by using the power analysis method: 0.05 alpha value, 1 estimated sigma value, and 75% of power detection. All data are expressed as mean ± SEM. Normal distribution was tested with d’Agostino and Pearson omnibus, Shapiro–Wilk, and Kolmogorov–Smirnov normality tests. If at least one of them was passed, statistical analysis was performed using parametric statistical analysis. Otherwise non-parametric Mann–Whitney or Kruskal–Wallis or Dunn’s tests were used. Before pairs of comparisons we performed the *F* test to compare variances. In experiments with normal distribution statistical analysis were performed using the unpaired two-sided Student’s *t*-test (95% confidence), one-way ANOVA with the Tukey’s as post hoc tests, two-way ANOVA with the Bonferroni’s post hoc test as appropriate and indicated in the figure legends. Values of *p* < 0.05 were considered as statistically significant. Grubbs’ test was performed to determine the significant outlier values. All experiments in this study were blinded and randomized by blocks of animals. All mice bred for the experiments were used for preplanned experiments and randomized to experimental groups. Data were collected, processed, and analyzed randomly. The experimental design and handling of mice were identical across experiments. Littermates were used as controls with multiple litters (3–5) examined per experiments.

## Data Availability

NetCal software is freely available at: http://www.itsnetcal.com/.
